# Sphingolipid-Transporting Proteins as Cancer Therapeutic Targets

**DOI:** 10.3390/ijms20143554

**Published:** 2019-07-20

**Authors:** Doaa Samaha, Housam H. Hamdo, Max Wilde, Kevin Prause, Christoph Arenz

**Affiliations:** 1 Institute of Chemistry, Humboldt-Universität zu Berlin, Brook-Taylor-Strasse 2, 12489 Berlin, Germany; 2 Depatment of Pharmaceutical Chemistry, College of Pharmacy, Helwan University, Cairo 11795, Egypt

**Keywords:** lipid transfer proteins, sphingolipids, lipid trafficking, sphingolipid transporters, transport inhibitors

## Abstract

The understanding of the role of sphingolipid metabolism in cancer has tremendously increased in the past ten years. Many tumors are characterized by imbalances in sphingolipid metabolism. In many cases, disorders of sphingolipid metabolism are also likely to cause or at least promote cancer. In this review, sphingolipid transport proteins and the processes catalyzed by them are regarded as essential components of sphingolipid metabolism. There is much to suggest that these processes are often rate-limiting steps for metabolism of individual sphingolipid species and thus represent potential target structures for pharmaceutical anticancer research. Here, we summarize empirical and biochemical data on different proteins with key roles in sphingolipid transport and their potential role in cancer.

## 1. Introduction

Lipids are essentially defined by their insolubility in water. In particular, membrane lipids, such as sphingolipids, can be transported in biological systems only within one membrane or between two different membranes. Since many processes controlled by sphingolipids are separated spatially, the transport of these molecules is of particular importance. The most obvious example is the spatial separation of processes of de novo sphingolipid biosynthesis. In contrast to degradation of sphingolipids, which is mostly catalyzed by soluble enzymes, all enzymes involved in the biosynthesis of sphingolipids are membrane-bound. Starting with serine-plamitoyl transferase (SPT), ceramide is synthesized in four steps within the cytosolic leaflet of the ER membrane. The synthetic steps downstream of ceramide, leading to formation of sphingomyelin, glucosylceramide, and more complex glycosphingolipids (GSL) take place at different localizations of the Golgi apparatus (GA). Therefore, there is much to suggest that the transport of intermediates between the different membranes is an essential component of sphingolipid biosynthesis. In addition, previous studies have shown that the function and activity of individual sphingolipid species is dependent on their specific subcellular or extracellular locations. This suggests a closer examination of the underlying transport processes.

In classical medicinal chemistry, it is a recognized fact that, in particular, enzymes that catalyze the rate limiting steps are well-suited pharmacological targets. The characterization of enzymes and their conversion rates, however, is usually carried out in vitro in cell lysates or even with purified enzymes. It is therefore obvious that the importance of sphingolipid transport proteins as potential bottlenecks of sphingolipid metabolism has not yet been sufficiently appreciated. Given the importance of sphingolipid metabolism as a potential pharmacological target in cancer, in this review, we would like to highlight proteins that are specifically involved in sphingolipid transport even if, in view of their recent discovery, only limited epidemiological data is available.

Transport processes and the proteins controlling these processes are emerging as new components of sphingolipid function with potential roles as pharmacological targets in the fight against cancer.

To understand trafficking of sphingolipids, various methods of lipid transport must be considered. These are also the subject of some current and more detailed overview articles [1,2]. Here, the most important principles of lipid transport are briefly mentioned before we go into specific transport routes and the enzymes that make them possible. According to the fluid mosaic model, lipids (and proteins) are more or less evenly distributed within a membrane layer like in a two-dimensional fluid. This model was later extended by the model of lipid rafts [3]. These are most easily summarized by the assumption that there are domains consisting of rather rigid and ordered lipids and proteins that float like rafts within the fluid membrane. Depending on their size, these domains can induce receptor clustering, suggesting a role as signaling platforms [4]. This model has been subject to debate and some modified models are being discussed [5,6]. Generally, it is assumed that these platforms are highly dynamic and that clustering can be triggered through different stimuli, which requires a directed lateral movement of membrane proteins and their associated lipids or vice-versa. So far, however, little is known about mechanisms underlying lateral transport of lipids. The other way of intramembrane transport, transversal transport from one sheet of the lipid bilayer to the other, also called flip-flop, may proceed in two different fashions. For lipids having small and not very polar head groups, a spontaneous flip-flop might be possible. In such cases, it is obvious, that flip-flop is essentially passive and follows concentration gradients. Lipids, which cannot undergo spontaneous flip-flop require an enzyme with flippase activity for transversal transport [7]. In such cases, the transport can oppose existing concentration gradients. Among the sphingolipids discussed in this review, only ceramide is able to undergo spontaneous flip-flop [8]. 

**Figure 1:**
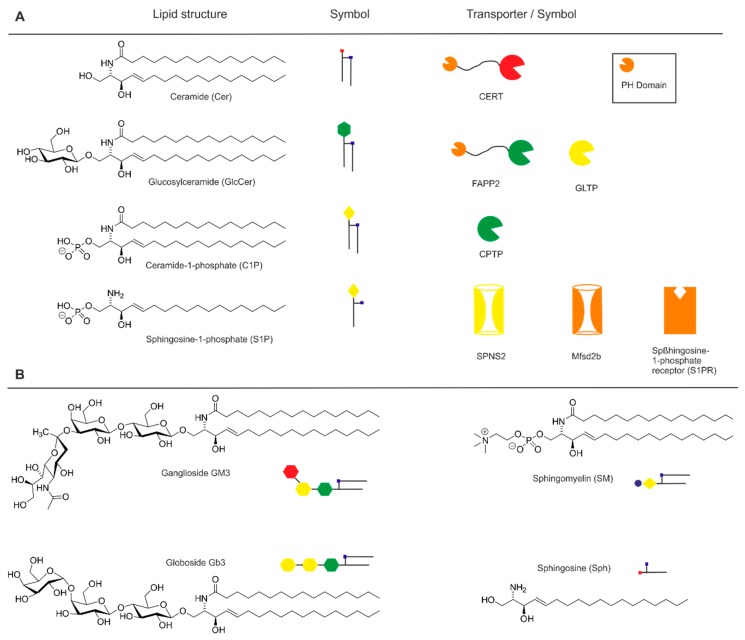
Sphingolipids and their specific transporters: (**A**) Structure of sphingolipids together with specific transporters and their symbols used in this review. (**B**) Structure of further important sphingolipids mentioned in this review.

Last, but not least, for transport of sphingolipids between distinct membranes there are again two opposing mechanisms known. The first mechanism, vesicular transport, starts with the budding of parts of the donor membrane via vesicle fission and ends with vesicle fusion with the acceptor membrane. This process is highly regulated by proteins and is dependent on ATP. In this process, a number of different lipids is usually transferred at the same time. The nonvesicular transport of lipids is mediated by more or less mono-specific lipid transporters that lift a distinct lipid out of the donor membrane for binding it within a hydrophobic cavity. After transport to the acceptor membrane, the cargo is discharged and the transporter can accept another cargo molecule for transport. This mechanism applies to the so-called lipid transport proteins (LTP), some of which (that are specific for sphingolipids) will be reviewed in this article. Although the sphingolipid transport proteins are all soluble proteins, it seems that an efficient transport of lipids requires the existence of membrane contact sites (MCS), in which different membranes come very close to each other [9,10]. The sphingosine-1-phosphate (S1P) transporters, that are also reviewed, substantially differ from the LTPs. While the substrates of LTPs are definitively membrane-bound, this does not necessarily apply to S1P. This lipid differs from the more complex sphingolipids by its higher polarity and higher solubility in water. Although S1P will bind to membranes, it is also relatively soluble and its critical micelle concentration was found to be 12 µM [11], which is well above its minimal bioactive concentration. It is therefore possible that proteins enabling the transport of this lipid through membranes are more like channels rather than being related to the LTP type of transporters in terms of mechanism. However, both types of transporters share the property that they crucially control the transport and thus the biological function of sphingolipid intermediates. In this review, the different known sphingolipid transport proteins are described with respect to their potential as targets for anticancer drugs. Figure 1 summarizes the structures of the preferred or exclusive sphingolipid substrates together with the names of the proteins that transport them. In addition, symbols are introduced for each lipid and transporter, which are referred to below.

## 2. Ceramide Transfer Protein (CERT)

The ceramide transport protein (CERT) is a protein that transports ceramides in a nonvesicular manner from endoplasmic reticulum to Golgi membranes, thereby influencing the metabolism, cellular concentration, and biological activity of this lipid [12]. When discovered, it was the first specific sphingolipid transport protein, making it a prototype for this type of transporter. Ceramide is a central component in sphingolipid metabolism and functions as a precursor for downstream sphingolipids and glycosphingolipids of higher complexity. Its concentration is controlled by more than 11 different enzymes [13]. It is synthesized in three separate pathways, which include sphingomyelin cleavage, de novo synthesis, and the salvage pathway [13]. The latter is a recycling pathway, fed by the degradation product sphingosine and by external sphingolipids. Ceramide has been implicated in several biological roles, particularly in the induction of apoptosis [13,14,15,16]. Ceramide levels are elevated following various triggers such as ultraviolet light, cytotoxic agents, ionizing radiation, or tumor necrosis factor alpha (TNF-α) [17]. In cancer, the lipid has been reported to be the main regulator of chemotherapy-induced cell death triggered by compounds, such as taxane, through induction of apoptosis [18]. Thus, several approaches to cancer chemotherapy are pharmacological manipulation of sphingolipid metabolism aiming to enhance cell ceramide as a proapoptotic molecule. 

CERT was identified by functional rescue experiments, showing that it is responsible for trafficking of ceramide from endoplasmic reticulum to Golgi independent of vesicular transport, but dependent of ATP [12]. During de novo synthesis of the membrane lipid sphingomyelin, the most abundant sphingolipid, this intermembrane translocation is the rate-limiting step. CERT is derived from the COL4A3BP gene (as annotated), which encodes two alternatively spliced proteins, the 624 and 598 amino acid isoforms, mostly termed as CERTL and CERT. The shorter splicing isoform is identical to GPBPDΔ26, which was originally termed Goodpasture antigen-binding protein (GPBP) [19]. CERT is ubiquitously present inside the cell. CERTL, its longer splice-variant is a nontypical serine/threonine kinase, which is partially secreted outside the cell, where it exists in solution or in a membrane-associated form [20]. Both isoforms are capable of transferring ceramide between cellular membranes [12,21]. The CERT protein is a soluble protein and comprises a set of characteristic domains and motifs: Starting from the amino terminus, a pleckstrin homology (PH) domain, ~120 amino acids in length, mediates binding to phosphatidylinositol 4-phosphate (PI4P) and therefore directs the CERT to the PI4P enriched Golgi complex [12]. It has been shown that the transfer process itself is not ATP dependent, but it has been speculated that the ATP dependence in situ may be essential to keep PI4P in a phosphorylated form. The PH domain is followed by the so-called middle region, ~250 amino acids in length, containing motifs that have been speculated to play a role in a potential homo- or hetero-oligomerization [19]. Furthermore, this region harbors the so-called FFAT motif, which docks the protein to the ER membrane via its interaction with the VAP protein (vesicle-associated membrane protein-associated protein) [22]. Finally, the C-terminus is formed by the START domain (steroidogenic acute regulatory protein), consisting of about 230 amino acids. The START domain is able and sufficient to bind and transfer ceramide in vitro, between donor and acceptor vesicles. The CERT protein is regulated by phosphorylation at multiple sites and phosphorylation generally attenuates the transfer process [23,24].

CERT is essential for embryogenesis. In CERT mutant embryos, sphingomyelin and plasma-membrane ceramides are reduced, while ceramide levels in ER and mitochondria are elevated, leading to severe mitochondrial dysfunction [25]. Interestingly, in the mutant embryos, the cell cycle was arrested, but without increased apoptosis, no growth arrest was reported in different cell lines with depleted CERT function. In contrast, CERT knock-out did not lead to ceramide accumulation in primary mouse embryonic fibroblasts (MEFs), neither in the ER nor in the mitochondria. However, higher concentrations of hexosylceramides have been described in these cells, suggesting a compensatory reaction of the cells to avoid high concentrations of cytotoxic ceramide in the ER. Nevertheless, substantial morphologic defects in the mitochondria and other organelles have been observed in the mutant MEFs.

### 2.1. CERT and Cancer

Swanton and colleagues showed that downregulation of CERT resulted in sensitization of tumor cells to various cytotoxic compounds by enhancing ER stress [26]. The authors used RNA interference (RNAi) screening in different tumor cell lines (MDA-MB-231/ breast cancer, HCT-116/colorectal cancer, and A549/non-small cell lung cancer) to evaluate all protein kinases and enzymes involved in ceramide metabolism, regarding their contribution to either resistance or sensitivity to chemotherapeutics such as paclitaxel. Paclitaxel belongs to the taxane group of anticancer drugs, which, through stabilization of microtubules, causes mitotic arrest and ultimately apoptosis. The authors identified a set of genes that confer resistance to paclitaxel, some of them being also implicated in mitotic spindle formation checkpoints. Knockdown of the related mRNAs potentiated the arrest of mitosis and induced initiation of endoplasmic reticulum stress, resulting in increased sensitivity to taxane. Among the different transcripts, COL4A3BP (CERT) triggered the most significant sensitization to taxane when it was knocked down. Indeed, CERT knock-down sensitized all types of cells to paclitaxel and additionally HCT-116 cells to 5-FU, MDA-MB-231 cells to doxorubicin, and A549 cells to cisplatin. Moreover, in drug-resistant cell lines as well as in ovarian cancer residual tumors after paclitaxel therapy, the authors identified an enhanced expression of CERT. Therefore, CERT could be a pharmacological target in cancers resistant to chemotherapy. Finally, HPA-12, a small-molecule inhibitor of CERT, was effective in elevating ER stress, supporting the hypothesis that ER stress is inversely correlated with CERT-mediated clearance of ceramide from the ER in the respective cell lines [26]. In another study, the same authors showed that among others COL4A3BP is a suitable marker for paclitaxel treatment outcome in therapy of triple-negative breast cancer [27]. Later, it turned out that ER stress induction is not the only contribution for CERT inhibition-mediated sensitization of cells to anticancer drugs, but also chromosomal instability (CIN) [28]. Furthermore, the sensitization of cells to chemotherapy following CERT depletion comes along with an increased expression of Lysosome-associated membrane protein 2 (LAMP2), which in turn leads to increased autophagosome-lysosome flux [28]. The authors provided evidence that the silencing of CERT resulted in decreased ceramide transport and thus increased levels of ceramides, even compared to paclitaxel-treated cells and the combination of CERT knock-down and paclitaxel treatment resulted in a synergistic elevation of the total ceramide levels. 

Despite these results, the suitability of CERT as a potential pharmacological target molecule in cancer cells is not always given and the story is not as simple as it may seem. One study provided evidence that CERT expression in triple-negative breast cancer (TNBC) and metastatic prostate cancer was decreased [29]. CERT obviously contributes to cell survival or cell death in more than one manner and a lowering of CERT expression can also provide advantages to tumor cells. This may also match with a recent study, which revealed a novel function of CERT and CERTL in the classical innate immune response, suggesting CERTL’s participation in apoptotic cell clearance [30]. CERT downregulation may be a way for the tumor cells to attenuate complement activation and thus evade the immune system.

### 2.2. CERT Inhibitors

**Figure 2:**
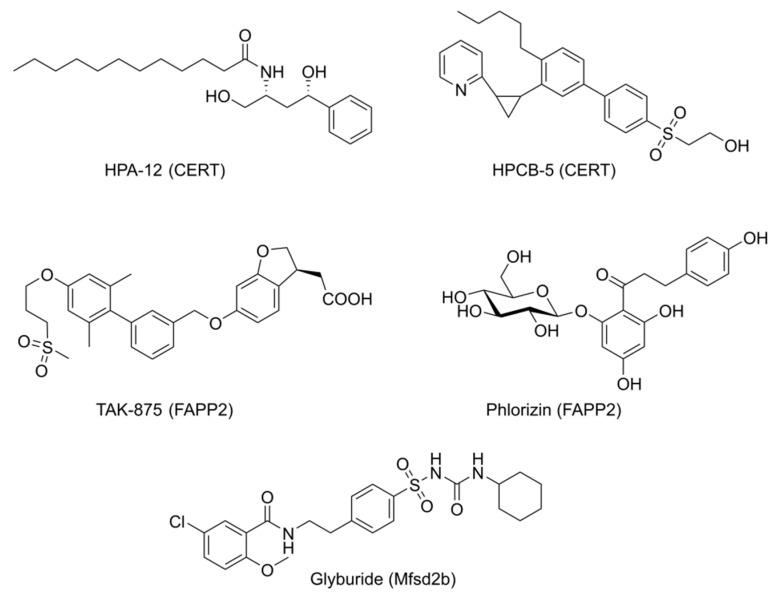
Known inhibitors of sphingolipid transfer or transport proteins.

The first and most prominent CERT inhibitor, HPA-12, was identified to inhibit ceramide trafficking prior to the identification of CERT [31]. It is structurally related to ceramide and shows potent cellular activity at concentrations between 0.1 to 2.5 µM (Figure 2). Since then, various syntheses and derivatives of HPA-12 have been published [30,32]. Very recently, the first not ceramide-related CERT inhibitor, HPCB-5, was identified by a virtual screening approach [33]. Its cellular potency is very similar to that of HPA-12.

## 3. FAPP2

FAPP2 or human phosphatidyl-4-phosphate adapter protein 2 is a protein essential for trans-Golgi network (TGN) to plasma membrane vesicular transport of glucosylceramide [34]. The protein is encoded by the PLEKHA8 gene, giving rise to 519 amino acids. It comprises a pleckstrin homology (PH) domain with 93 residues with two specific binding sites for PI4P. The PH domain is linked by a stretch of ~200 amino acids to the C-terminal glycolipid transfer protein homology (GLTPH) domain of 212 amino acids.

In accordance with the existence of the GLTPH domain, the protein is able to selectively transfer glucosylceramide (GlcCer) from cis Golgi to the TGN [34]. GlcCer is the precursor for the biosynthesis of more complex GSL, which takes place on the luminal sheet of late Golgi membranes. Recently, the structure of FAPP2 was resolved and provided insight into the binding mode [35]. The selectivity for the ceramide moiety was found to originate from highly conserved His and Asp residues of the recognition center that bind the amide group of ceramide. A group of four hydrophobic residues plays a sensor role in sphingolipid chain length determination. The recognition center, together with so-called ID loop elements, determine the sugar head-group specificity for substrate glycosphingolipids.

Indeed, knock down of FAPP2 leads to significantly reduced concentration of complex GSL. A more detailed study specified that FAPP2 is essential for the synthesis of glycosphingolipids of the globo-series, while the ganglioside GM3 and other gangliosides, which are also derived from GlcCer, but in earlier Golgi membranes like the cisternae, are synthesized independently of FAPP2 [36]. Still, many aspects of FAPP2 action and the underlying mechanisms await further clarification. Since the synthesis of the complex GSL is located at the luminal side of the Golgi, it is yet unknown how the trans-bilayer movement is facilitated. This could be mediated by a flippase enzyme, catalyzing the trans-bilayer flip-flop, but such a factor is still elusive [37]. An alternative model proposes the retrograde transport of GSL from Golgi to ER, where they can be wrapped into vesicles, which can then be transported anterograde and fuse with the Golgi [38]. However the latter model may not explain the differences seen for the synthesis of globosides and GM3. Moreover, it is still not finally clear whether the role of FAPP2 in TGN vesicle budding is a consequence of its limiting role in GSL synthesis. The fact that the Trp407Ala variant of FAPP2, which is devoid of GlcCer binding and glycolipid transfer activity, shows a diminished TGN to plasma membrane vesicular transport when expressed in FAPP2−/− cells, points in this direction [37]. On the other hand, FAPP2 has an independent ability to bend membranes and to form tubular structures in model membranes. This feature also seems to be essential for TGN vesicle budding. Another interesting hint for the importance of FAPP2 comes from its role in HCV infection [39]. A viral protein was shown to activate an ER-derived PI4-kinase, leading to elevated levels of PI4P at the HCV replication complex. As a result, FAPP2 is hijacked (via its PH domain) to transport GSL to the HCV replication complex. Knock-down of FAPP2, in contrast, led to reduced viral replication and infectivity.

### 3.1. FAPP2 in Cancer

The data for a role of FAPP2 in cancer is rather limited. An early study described induction of apoptosis in colon cancer carcinoma cells after incubation with ribozymes targeting FAPP2 in the presence of Fas agonistic antibody [40].

In a recent publication, FAPP2 was remarkably upregulated in colon cancer samples compared to adjacent tissues in 52 out of 90 patients. In 20 patients, FAPP2 was unchanged and in 18 patients, FAPP2 was downregulated [41]. In FAPP2 upregulated cancer tissues, disruption of FAPP2 by CRISPR/Cas9 led to attenuated cell growth and colony formation. The tumorgenicity of FAPP2 downregulated xenografts in a nude mice model was also decreased. Further mechanistic evaluation showed that FAPP2 affects Wnt/beta-catenin signaling [41]. However, further research will be needed to confirm the potential of FAPP2 as a target for cancer therapy.

### 3.2. FAPP2 Inhibitors

Inhibitors of FAPP2 have so far only been described in one patent [42]. The compound Phlorizin leads to a notable decrease of the FAPP2 transfer activity. TAK-875, a selective agonist of GPR40 (Free fatty acid receptor 1), was identified as a FAPP2 inhibitor (Figure 2). Since FAPP2 downregulation leads to a concomitant decrease in glycosphingolipids, such inhibitors may be promising tools for substrate deprivation therapies in glycosphingolipid storage diseases [43].

## 4. Glycolipid Transfer Protein (GLTP)

Glycolipid transfer protein (GLTP) is 24 kDa soluble cytosolic lipid binding protein with a putative role in nonvesicular glycolipid trafficking. In vitro it has been shown to catalyze intermembrane translocation of various glycolipids and glycosphingolipids (GSLs) [44,45]. GLTP’s in vitro functional properties and its structural features are subject of excellent reviews [46]. The X-ray crystal structures of GLTP with and without bound substrates have been resolved and revealed yet unknown folding motifs. Due to its novel architecture, GLTP has been considered the prototype of a new family of lipid-binding proteins [46,47]. The protein adopts a previously unknown all α-helical structure, which is arranged in a ‘sandwich motif’ to form a single glycolipid-binding pocket [45]. GLTP binds both ceramide-derived glycosphingolipids as well as glycerol-based glycolipids. However, the mono- or oligosaccharide-part must be linked to the lipid backbone via a beta-glycosidic bond. The rate-limiting step in GLTP-mediated transfer reactions was found to be the formation of the GLTP-substrate complex and its release from the donor membrane, which is consistent with a shuttle/carrier mode of action [48]. GLTP has no PH domain and its in vivo target membranes are uncertain. The high affinity of GLTP for GlcCer on the one side, together with a low affinity for membranes on the other side, suggests that GLTP may also function as a dynamic reservoir for GlcCer which could deliver its cargo when and where it is needed. In vitro experiments showed that the uptake of glycolipids by GLTP is a function of the molar fraction of the lipid within the donor membrane. It is likely that also in vivo the donor and acceptor membranes will be defined according to this principle [49]. 

While orthologous forms of GLTP in plants and fungi play a role in programmed cell death, the function of the protein in mammals is largely unclear [50,51]. At least, the depletion or overexpression of GLTP failed to induce apoptosis in mammalian cell lines, in agreement with earlier GLTP overexpression data [52]. However, when human GLTP was overexpressed in HeLa or HEK-293 cells, significant alterations in cell shape were observed [53]. In contrast to the tubule-forming activity of FAPP2, this feature depends on the presence of a functional glycolipid binding site [54]. Overexpression of GLTP increases glucosylceramide (GlcCer) and globo series (Gb3) content, while RNAi-mediated knock-down of GLTP leads to reduced Gb3 concentration in HeLa cells [50]. 

In cells with elevated levels of GlcCer, the GLTP expression was upregulated, while a decrease in GlcCer concentration, caused by glucosylceramide synthase knock-down, resulted in significantly reduced GLTP expression levels. Therefore, GLTP has been suggested to act as a sensor for newly synthesized glucosylceramide (GlcCer) with a putative regulatory role in interorganelle glycosphingolipid redistribution [50,54]. 

### 4.1. GLTP and Cancer

GSLs and other glycolipids play important roles in adhesion processes at the cell surface [55] as well as in neurodegeneration and cell death. Especially in colon cancer, initiation and progression is strongly associated with altered levels in glycosphingolipids [56]. Due to its proposed role as molecular transporter of GlcCer and other glycosphingolipids to the plasma membrane [57], and due to its potency to induce changes in cell shape changes, GLTP appears to be involved in progression and malignancy of cancer. 

A functional study on miR-196B, a microRNA upregulated in colon cancer, showed its ability to suppress FAS-mediated apoptosis [58]. Later, three direct targets of miR-196B were identified and validated: HOXA5, HOXB6, and GLTP. Indeed, a comparison with adjacent nontumorous tissues showed that GLTP protein expression was lowered in colorectal cancer tissues [59]. Moreover, very recently, the overexpression of human GLTP was shown to induce cell death via necroptosis in certain colon cancer cells [60]. GLTP overexpression resulted in cell cycle arrest and a dormant state for HCT-116 cells and normal colonic cells. These cells showed slightly elevated ceramide and unchanged S1P levels. In HT-29 cells, the same treatment resulted in a cell cycle arrest, which was accompanied by only moderately lowered ceramide levels together with a drastic reduction in S1P levels and finally a shift in the so-called ‘sphingolipid-rheostat’ leading to necroptotic cell death [60]. Moreover, it was found that the resistance of cells to undergo cell death upon GLTP overexpression [53] was associated with low expression levels of receptor-interacting protein kinase (RIPK-3) [60,61]. 

### 4.2. GLTP Inhibitors

To the best of our knowledge, no small molecule inhibitors of GLTP are known. 

## 5. Ceramide-1-Phosphate Transport Protein (CPTP)

CPTP was originally termed GLTP1, due to its GLTP-related structure [51]. Later, it was found that instead of glycolipids the protein is specific for ceramide-1-phosphate and it was renamed to ceramide-1-phosphate transfer protein, CPTP [62]. The structural similarity of CPTP to GLTP was confirmed by X-ray structural analysis. CPTP contains a positively charged surface pocket. The C1P head group is recognized by positively charged amino acids and the K60A or R106L variants of the protein show virtually no C1P transfer activity [62]. The glycerol-lipid analog of C1P, phosphatidic acid, is not transferred, but the hydrophobic pocket suggests acceptance of a wide range of different chain lengths. The protein is mainly localized to the cytosol, but is also found associated with perinuclear membranes, the plasma membrane, and in the nucleus. Knock down of the CTPT by RNAi elevates intracellular levels of C1P of different chain lengths mainly in the TGN and leads to partial fragmentation of the Golgi apparatus and these effects were not rescued by overexpression of mutants devoid of transfer activity. It is likely that CPTP transfers C1P, after its synthesis in the TGN, to the plasma membrane and other compartments. However, the structural analysis of the protein did not reveal any domains for targeted recognition, such as a PH domain [62]. It is thus still unclear whether the protein can mediate a directional transfer of C1P. However, recently, it was shown that the protein and its *Arabidopsis* orthologue ACD11 are directly and specifically stimulated by phosphatidyl serine (PtdSer), which provides evidence for a head-group-specific interaction site on the protein’s surface [63].

### 5.1. CPTP and Cancer

Ceramide-1-phosphate, C1P, has opposite activity to ceramide and acts as a mitogenic and prosurvival lipid [64]. Intracellularly produced C1P is also a mediator of inflammatory processes and via PLA2 stimulation and increased production of eicosanoids, thereby contributing to chronic inflammation. In addition, extracellular C1P has been reported to stimulate motility of cells, including pancreatic cancer stem cells [65]. In their initial study, Brown and colleagues showed that siRNA-mediated downregulation of CPTP increased cellular C1P, while—obviously as a result of concomitantly decreased plasma membrane C1P—the phospholipase A2 (PLA2)-mediated release of arachidonic acid and downstream eicosanoids is stimulated. Along this line, the *Arabidopsis* orthologue of CPTP, ACD11 (accelerated cell death 11) was shown to elevate prodeath sphingolipids when mutated [66].

Therefore, CPTP is regarded as a potential biomarker in cancer [67]. A more recent study showed that CPTP is a regulator of autophagy and inflammasome activation [68]. A knockdown of CPTP induced autophagy in epithelial cells by up to ten-fold and activated Caspase1 and elevation of cytokines. 

Recently, it was shown that CPTP is a direct target of miR-328 [69]. Upregulation or delivery of this miRNA and thus downregulation of CPTP sensitizes non-small cell lung cancer cells to radiotherapy or colorectal cancer cells to chemotherapy. Also, a recent meeting abstract suggests that CPTP (and GLTP) regulates neoplastic progression of colon and breast cancer cells [70]. It seems that CPTP expression levels are often altered in various cancer biopsies, but a conclusive study in this direction is still elusive and more solid evidence for CPTP being a promising anticancer target is needed.

### 5.2. CPTP Inhibitors

There are currently no known small molecule inhibitors of CPTP. 

## 6. Sphingosine-1-Phosphate Transporters

Sphingosine-1-phosphate is a potent bioactive molecule involved in signaling events. It is produced inside cells from sphingosine by sphingosine kinase 1 or 2 (SphK1/2). After its transport to the outer leaflet of the plasma membrane, it can act in an autocrine or paracrine manner on a number of specific S1P receptors (S1PR1-5), which are G-protein coupled receptors of the plasma membrane. By binding to these receptors, S1P can trigger a plethora of downstream biological effects that are “prosurvival” such as cell migration. S1P signaling via S1PRs is involved in many pathologies, including autoimmune diseases and cancer [71,72]. For this so-called “inside-out signaling”, there must be defined mechanisms of transport of S1P outside the cells. Indeed, it is a well-established fact that blood and body fluids display high concentrations of S1P, in contrast to low levels in tissues. Besides the ABC transporters, which have been reported to play some role in S1P evasion of cells, there have recently been two distinct specific S1P transporters described, SPNS2 [73] and Mfsd2b [74]. Results from knock-out mice suggest that each of the transporters accounts for roughly 50% of plasma S1P levels. In contrast, mice with genetic knock-outs for different ABC transporters did not show altered S1P levels. While Mfsd2b is highly abundant in the plasma membranes of red blood cells, platelets, and in spleen and bone marrow [74], SPNS2 is expressed mainly in epithelial cells and in other cells and tissues, except bone marrow and erythrocytes and platelets [73]. Based on the current knowledge, each transporter is discussed individually. 

## 7. Spinster Homolog 2 (SPNS2)

Spinster homolog 2 is a member of a large superfamily of non-ATP-dependent organic ion transporters. SPNS2 was discovered as a S1P transporter from Zebrafish studies [75]. A mutation in this gene caused a block in the migration of cardiac precursor cells and a split heart phenotype, which mimicked the phenotype of an S1P-receptor 2 (S1PR2) knock-out. In the following, it was shown that overexpression of SPNS2 increased plasma S1P and dihydrosphinganine-1-P levels, but not levels of sphingosine. Downregulation conversely led to decreases in the plasma levels of S1P. In contrast to zebrafish, knock-out mice are viable and appear normal, except organ-specific defects like deafness or vascular and retinal neurologic defects probably due to defects in the migration of neurons during retina development. These defects were observed in global S1P knock-outs, but not if the knock-out was specific for different tissues. This clearly shows that SPNS2 has a dual role in regulating systemic but also local and tissue specific S1P levels. As mentioned above, the global SPNS2 knock-out reduced the plasma S1P to about 60% and to the same extent as endothelial cell specific knock-out. Interestingly, SPNS2 systemic or endothelial cell-specific knock-out lead to lymphopenia [76], in contrast to the Mfsd2b knock-out, which results in a similar decrease in plasma S1P. This indicates that S1P produced from endothelial cells has a specific role in regulation of lymphocyte trafficking [77].

### 7.1. SPNS2 and Cancer

Due to the power of S1P as a signaling molecule, the role of S1P transporters in cancer is worthy of in-depth investigation [78]. In a first study, SPNS2 was downregulated in a small cohort of human lung tumors and overexpression of SPNS2 in non-small cell lung carcinoma cells led to increased S1P secretion, decreased cell migration, and induction of apoptosis [79]. SPNS2 knock-down also led to decreased EGF-mediated invasion of HeLa cells [80]. Interestingly, in this study, which showed that the mitotic transition was dependent on SPNS2 and the S1PRs, the effects were not blocked by an anti-S1P antibody, suggesting that there might be endogenous secretion pathways for SPNS2 as well. 

Apart from these results for SPNS2 in tumor cells, the most striking result highlights the importance of healthy tissue SPNS2 for metastasis. In a recent study, more than 800 randomly selected knock-out mice were tested for their susceptibility to metastatic colonization of the lung. Among all mice, those having the SPNS2 gene knock-out showed the lowest metastatic burden [81]. Interestingly, the low level of metastasis was not due to an inability of cancer cells to invade the lung, but instead due to an unfavorable environment for metastatic colonization in the lung. Despite the lymphopenia seen in the SPNS2 knock-out mice, the level of natural killer cells in the lungs was increased, together with the T cell activities of CD4+ and CD8+ T cells, which all contribute to reduced metastasis. These results strongly suggest targeting of SPNS2 as a strategy to reduce metastasis after surgical resection of tumors.

### 7.2. SPNS2 Inhibitors

To our knowledge, there are no known small molecule inhibitors of SPNS2.

## 8. Mfsd2b

Since the knock-out of SPNS2 resulted only in a reduction of plasma S1P to about 60%, it was clear that there must be another source for plasma S1P [78]. Very recently, Mfsd2b was identified as an ATP-dependent S1P transporter in erythrocytes and platelets [74,82]. Knock-down of this transporter reduced plasma S1P to about 50%. Therefore, it is now believed that SPNS2 from endothelial cells and Mfsd2b from red blood cells and platelets contribute to most if not all plasma S1P in equal portions, respectively.

### 8.1. Mfsd2b

Up to now, there is no direct data for the recently discovered Mfsd2b in cancer. However, the known role of S1P in cancer suggests that an investigation of this topic would be highly worthwhile. It is known that many tumors overexpress sphingosine kinase 1, but trials with PF-543, a potent inhibitor of this enzyme, were unsuccessful. In contrast, experimental therapy with Sphingomab, an S1P antibody which can “soak” plasma S1P, showed promising results in a mouse model of renal cell carcinoma. The antibody also shows suppression of lung metastasis, which matches the results from SPNS2 knock-out leading to decreased systemic or plasma S1P levels. 

### 8.2. Mfsd2b Inhibitors

Mfsd2b was found to be expressed in the erythroid cell line MEDEP-E14. In these cells, Mfsd2b was inhibited by very high amounts of glyburide (Figure 2). The amount of extracellular NBD-labeled S1P was reduced to 50% compared to untreated cells in the presence of 500 µM glyburide [82].

## 9. Conclusions

**Figure 3:**
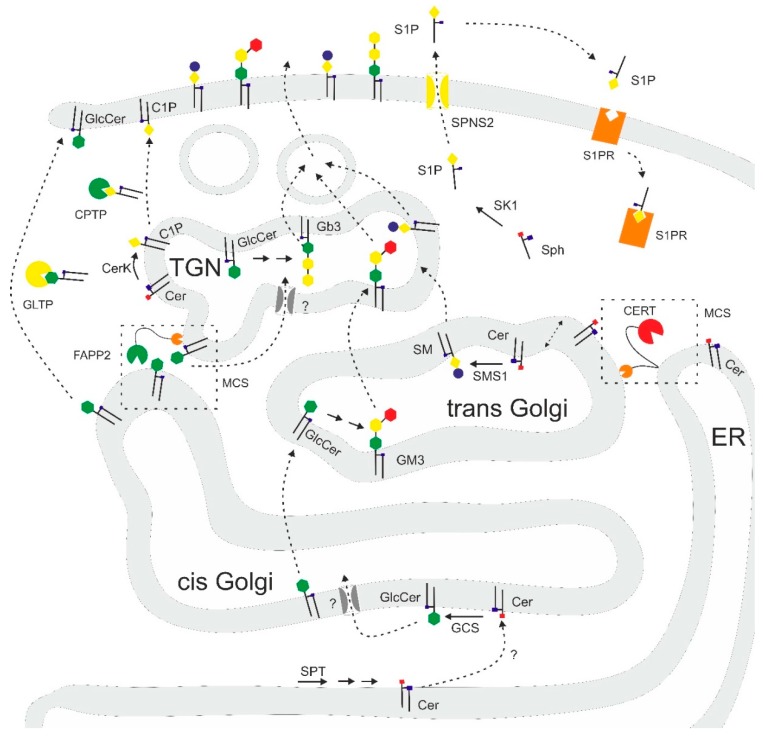
Overview of sphingolipid biosynthesis-associated trafficking (simplified). Solid arrows: enzyme mediated reaction; dashed arrows: transport event; SPT = serine palmitoyl transferase; GCS = Glucosylceramide synthase; SMS1 = sphingomyelin synthase 1; CerK = ceramide kinase; SK1 = Sphingosine kinase 1; MCS = membrane contact site; TGN = trans Golgi network; ER = endoplasmic reticulum. Grey channels: not clearly defined transporters that likely include members of the ABC family of transporters like MDR1/p-glycoprotein.

Sphingolipid transfer proteins and sphingolipid transporters are recently characterized key components for sphingolipid metabolism and function. They are components of a complicated and finely balanced network of metabolic processes that can only take place at specific sites within cells or even organelles. These proteins thus provide the cell with an additional level of regulation that can possibly also be addressed by pharmacological intervention. A simplified overview of this network with the transport proteins discussed here is shown in Figure 3. At present, many aspects of intracellular sphingolipid transport are still unclear. For example, there is clear evidence that members of the ABC transporter family of proteins such as MDR1 or p-glycoprotein also play an important role as flippases for GlcCer and sphingomyelin, but the actual in vivo mechanisms await further elucidation [83,84].

Although not much is known about individual proteins and their roles in cancer, the preliminary results obtained so far are highly promising. However, it must be emphasized once again that most—if not all—data come from preclinical studies. Therefore, future systematic clinical trials are required. A similar situation also exists for small molecule inhibitors, which are often barely potent and selective, if they exist at all. We therefore hope that this review article helps to highlight this promising group of proteins and to stimulate further investigations. We believe that the data so far, together with the undisputed importance of sphingolipids in cancer, justify this.
